# Long‐term views on chronic kidney disease research priorities among stakeholders engaged in a priority‐setting partnership: A qualitative study

**DOI:** 10.1111/hex.12818

**Published:** 2018-08-15

**Authors:** Meghan J. Elliott, Joanna E. M. Sale, Zahra Goodarzi, Linda Wilhelm, Andreas Laupacis, Brenda R. Hemmelgarn, Sharon E. Straus

**Affiliations:** ^1^ Department of Medicine University of Calgary Calgary AB Canada; ^2^ Department of Community Health Sciences University of Calgary Calgary AB Canada; ^3^ Li Ka Shing Knowledge Institute St. Michael's Hospital Toronto ON Canada; ^4^ Institute of Health Policy, Management, and Evaluation University of Toronto Toronto ON Canada; ^5^ Hotchkiss Brain Institute University of Calgary Calgary AB Canada; ^6^ Canadian Arthritis Patient Alliance Toronto ON Canada; ^7^ Department of Medicine University of Toronto Toronto ON Canada

**Keywords:** chronic kidney disease, patient engagement, patient‐oriented research, qualitative research, research priorities

## Abstract

**Background:**

Patients and stakeholders are increasingly engaging in health research to help address evidence‐practice gaps and improve health‐care delivery. We previously engaged patients, caregivers, health‐care providers (HCPs) and policymakers in identifying priorities for chronic kidney disease (CKD) research.

**Objective:**

We aimed to explore participants' views on the research priorities and prioritization process 2 years after the exercise took place.

**Design:**

In this qualitative descriptive study, individual interviews were conducted and analysed using an inductive, thematic analysis approach.

**Setting/participants:**

Participants resided across Canada. We purposively sampled across stakeholder groups (CKD patients, caregivers, HCPs and policymakers) and types of engagement (wiki, workshop and/or steering committee) from the previous CKD priority‐setting project.

**Results:**

Across 23 interviews, participants discussed their research priorities over time, views on the prioritization process and perceived applicability of the priorities. Even though their individual priorities may have changed, participants remained in agreement overall with the previously identified priorities, and some perceived a distinction between patient and HCP priorities. They tended to balance individual priorities with their broader potential impact and viewed the prioritization process as systematic, collaborative and legitimate. However, participants acknowledged challenges to applying the priorities and emphasized the importance of communicating the project's outcomes upon its completion.

**Conclusion:**

Two years after engaging in CKD research prioritization, stakeholder participants remained in agreement with the previously identified priorities, which they felt reflected group deliberation and consensus. Rapport and communication were highlighted as key elements supporting effective engagement in research prioritization.

## 1 INTRODUCTION

Chronic kidney disease (CKD), characterized by persistently abnormal kidney function, affects up to 13% of the population globally^1^ and is associated with considerable morbidity, mortality and health‐care costs.^2^ However, there is a lack of high‐quality evidence to guide CKD treatment,^3^, ^4^ and even where evidence exists, it is not always translated into practice, thereby leading to evidence‐care gaps.^5^ One potential way to address such gaps and optimize delivery of patient‐centred CKD care is to engage research end‐users (ie patients, caregivers, clinicians and policymakers) in establishing the research agenda.^6^, ^7^ Because the priorities of those most impacted by research findings may not align with those of the research community, redirecting the focus of research to stakeholder‐identified priorities may enhance the relevance and uptake of evidence into practice.^6^, ^8^, ^9^

In 2015, we used the approach established by the James Lind Alliance (JLA) to identify the most important, unanswered questions about CKD management among CKD stakeholders, including adult patients with non‐dialysis CKD, their caregivers, and health‐care providers (HCPs) and policymakers involved in CKD care.^10^, ^11^ The final step of this internationally recognized process includes a 1‐day workshop where participants convene to discuss and rank the top 10 research priorities. To address the limitations of an in‐person meeting, such as travel and resource requirements, we conducted a randomized controlled trial comparing the traditional in‐person workshop to a novel, online wiki‐like platform for ranking the final priorities.^12^ Although there was some overlap between the two groups' top 10 priorities, findings from a post‐intervention survey indicated that wiki participants felt they were less able to express their views and contribute meaningfully.^12^ The priorities identified through this exercise, as well as those from a related JLA partnership for those with advanced CKD on or nearing dialysis, were used to inform all research projects within a national patient‐oriented kidney research programme in Canada.^13^

The impact of patient and stakeholder engagement on participants and research has not been fully assessed,^14^, ^15^ and existing reports have tended to focus on short‐term perceived impacts of engagement.^16^ Of those studies involving patients in research prioritization, none has examined participants' views on the priorities beyond the completion of the priority‐setting exercise. As research programmes and funding decisions increasingly address priorities identified through processes such as that of the JLA,^17^, ^18^, ^19^ it is important to understand how stakeholders who contributed to the research priorities consider them over the long term. In this qualitative study, we sought to characterize how stakeholder participants from a CKD priority‐setting project viewed the research priorities and the prioritization process 2 years after the exercise took place.

## 2 METHODS

### 2.1 Study design and setting

This study was guided by a qualitative descriptive methodology,^20^, ^21^ which allowed us to explore participants' experiences with the priority‐setting project and their individual approaches to determining CKD priorities. The full JLA process for this project has been described previously.^10^, ^12^ Participants included CKD stakeholders from across Canada. The steering committee (*n* = 13) met regularly over a 1‐year period to determine a priorities' shortlist, from which the final 10 priorities were ranked. In the final prioritization step, we compared a traditional, 1‐day, in‐person workshop held in Toronto, Canada, in June 2015 (*n* = 26) to a 2‐week, online, wiki‐like process (*n* = 27) for group ranking of the priorities.^12^ Eleven of the steering committee members attended the in‐person workshop (as participants or facilitators).

We observed principles of rigour in the design and conduct of this qualitative study, including suitability of the research question to qualitative research, transparency in our design and sampling, provision of sufficient support for our findings and clear integration and interpretation of the data.^22^ This project was undertaken as part of the lead author's graduate thesis, in which an individual living with a non‐CKD chronic condition (L.W.) collaborated as a supervisory committee member. L.W. has served in several patient‐oriented research and health policy capacities and provided input at several stages of this project, including suggesting modifications to study design (eg adapting interview guide and technique), reviewing final themes for coherence and offering interpretive insights into our findings, particularly as they related to implications for patients. L.W. participated in team meetings by telephone/online platform and reviewed all outputs from this project. She continues to play a role in disseminating concepts from this study through her involvement with patient advocacy organizations. Informed consent was obtained from all participants. The Research Ethics Boards of St. Michael's Hospital, the University of Toronto and the University of Calgary approved the conduct of this study.

### 2.2 Participant selection

For eligibility in the original priority‐setting project, English‐speaking, adult participants from a relevant CKD stakeholder group had to have had access to high‐speed Internet and high health literacy^23^ to meet the technical requirements of engaging collaboratively online. Fifty‐three participants from the original project were eligible for inclusion in this study (Appendix S1). Participants had taken part in one or more aspects of the project (steering committee, workshop or wiki) as a CKD stakeholder (patients with non‐dialysis CKD, caregivers, HCPs and policymakers). To identify unique perspectives and common patterns across varied roles in the project, we purposively sampled among all stakeholder groups and types of engagement.^20^, ^24^ Eligible participants were invited by email to participate in a telephone interview; those who resided in Toronto were given the option of a face‐to‐face interview. The use of both telephone and in‐person interviews in the same study yields similarly acceptable data and should not compromise the quality of the findings.^25^

### 2.3 Data collection

We conducted in‐depth, semi‐structured interviews to explore participants' views on CKD research priorities and experiences with research prioritization. All interviews were conducted by a single interviewer (M.J.E.). One week prior to their interview, participants were provided with the original shortlist of 30 CKD research priorities and the top 10 ranked priorities from the workshop and wiki groups. Participants were asked to reflect on these priorities and consider how they might rank them now. We referred to an analytic‐deliberative conceptual model for stakeholder engagement in research^26^ when developing our interview guide, which addressed their experiences with the priority‐setting project and their previous and current priorities for CKD research (Appendix S2). We pilot‐tested the interview guide with a qualitative researcher experienced in patient engagement and made minor revisions to it following each of the first three interviews (ie to the wording and ordering of questions, not the content of the interview guide). All interviews were audio‐recorded, transcribed verbatim and uploaded in NVivo 11 (QSR International Pty Ltd, Victoria, Australia) to facilitate data organization and coding. Demographic information (ie age, sex, province of residence, kidney function [patients/caregivers] and job title [HCPs/policymakers]) was collected for the purposes of summarizing our sample and contextualizing our findings.

### 2.4 Data analysis

Data collection and analysis took place concurrently. Guided by a thematic analysis approach,^27^ two researchers (M.J.E. and Z.G.) independently generated initial codes representing each expressed idea to systematically organize the data within and across transcripts. The researchers met after coding the first three transcripts to discuss the evolving coding scheme and again after coding every three to four subsequent transcripts to refine this scheme and discuss analytic insights. Three other members of the research team reviewed the first three transcripts to ensure relevant data were captured and offer preliminary insights. No new codes were identified beyond the first 10 transcripts, and subsequently, only minor adjustments to code definitions were made. We organized coded data extracts relevant to the research question into preliminary themes, which we then reviewed and refined to ensure clear connections among them and with the coded data. Developing findings were discussed among the larger research team, and direct quotes were highlighted to illustrate our findings. Data saturation was achieved when no additional relevant data were collected in the interviews. In this report, our use of the term “stakeholders” broadly refers to all stakeholder roles included in this study (ie patients, caregivers, HCPs and policymakers), unless otherwise specified.

## 3 RESULTS

Of the 53 eligible participants, 23 completed an interview (20 by telephone; 3 face to face) lasting approximately 1 hour. Of those who did not participate, 5 people declined, 4 could not be reached, and 21 did not respond to our email invitation. As we achieved data saturation and representation across stakeholder groups, we did not make further attempts at recruitment. Of the 23 participants, 8 were patients, 4 were caregivers, 8 were HCPs (ie nephrologist, nurse, dietician or pharmacist) and 3 were policymakers. Fourteen people had participated in the final in‐person CKD priority‐setting workshop (6 of whom were also on the steering committee), 7 had participated in the online wiki‐like platform, and 2 were only on the steering committee.

Participants' discussions of the CKD research priorities centred around three related concepts: (a) research priorities over time; (b) research prioritization process; and (c) application and applicability of priorities.

### 3.1 Research priorities over time

In discussing their priorities for CKD research at the time of the workshop/wiki, patients and caregivers reflected on elements of living with CKD, while HCPs tended to prioritize areas related to CKD care delivery. For example, one patient felt strongly about the “slowing the progression ones [priorities]” (ID22), as they related directly to his declining renal function, whereas one HCP “saw a lot of symptom type management being a big issue” (ID3) given the nature of his clinical work. Several participants highlighted a perceived distinction between the priorities of different stakeholders, suggesting that whereas health policy priorities seemed more relevant to clinicians/policymakers, those related to CKD diet, quality of life and alternative/complementary therapies may be more important for patients:

And I probably shouldn't have been surprised, but at the time I was a bit surprised at how passionate patients were about prevention, diet, and nutraceuticals and things like that, which, of course I hear in my clinic every day. They're always asking me about it, but as a [HCP and] researcher, I kind of downplay the relevance of those things. (ID14, HCP)

Despite this perceived difference, participants across groups tended to agree with the final priorities and in particular with the top‐ranked priority from both groups, “interventions to prevent the development and progression of CKD.” Some indicated that the final list fairly represented the group's discussion and what were “probably the most important questions” (ID19, HCP). Participants' overall agreement with the top 10 priorities supports the general stability of the group's priorities over time. Some indicated that while individuals' priorities may change over time, those for the “broad‐based population would [not] necessarily change” (ID23, patient). Further, several participants, such as this HCP, acknowledged an important limitation of priority‐setting exercises—that the final priorities may not reflect those of other individuals or in other contexts:

It's really asking select patients that actually are well enough to be here… So I walked away from it saying it's somewhat biased, because the patients that were there were the ones that really wanted to be there in the first place, and are they really the voice of the ones that aren't here? (ID3, HCP)

Patients whose CKD had progressed over the last 2 years described how their priorities for research had changed as a consequence of experiencing complications of advanced CKD. For example, one patient, who had since started dialysis, mentioned that she now considered kidney transplantation an important priority, whereas another described the increased significance he had since placed on symptoms. Although the priorities for patients and caregivers of those with stable CKD remained largely unchanged (“I have no different issues than I had five years ago” [ID10, patient]), their additional experience living with CKD may have influenced how they viewed the priorities. Similarly, HCPs and policymakers described how their priorities were responsive to subsequent experiences with CKD care delivery, awareness of new local/regional care issues, emerging evidence and funding issues. The following quotes support these long‐term views on the research priorities:

I think it [‘symptoms’ priority] was pretty important for me then, but it's even more so now because I have dealt with extreme fatigue and low energy, sleeping problems due to severe itchiness, which I know is a huge thing for patients with lower function. (ID17, patient)

One of the things that is most important to me now wasn't picked as one of the most important before [‘access to care’ priority]. Just because we're seeing more and more patients come out of our First Nation communities, and it's like an epidemic here now, so my top priority would be: How do you ensure patients receive care in those communities for their kidney disease? (ID13, policymaker)

### 3.2 Research prioritization process

When weighing the relative importance of the CKD research priorities, participants described balancing their own circumstances, concerns and needs (“It comes right down to what do I think is best for me” [ID21, patient]) with those of the others taking part in the prioritization exercise. Participants also considered the potential relevance of the priorities to the broader CKD population, including “what's going to be most effective for a lot of people” (ID4, patient) and “what would help everybody… not [what is] necessarily one person's specific experience” (ID7, caregiver). Several HCPs described how clinical encounters and/or inadequate evidence guiding CKD care influenced how they identified priority areas for research, such as one HCP for whom “a gap in knowledge for patient care” (ID3) was an important consideration. Similarly, HCPs commonly expressed that they more heavily weighed a priority that had potential to meaningfully influence patient care and strengthen its evidence base. As an illustration of this, one HCP said:

I think about what questions I get or what I deal with most in terms of patient issues. So that would be one. And then I also think, as a researcher, I would think about what is answerable or what is most answerable with a good answer rather than just contributing a little bit of information that won't move medicine and our care forward. (ID19, HCP)

When ranking the priorities, participants identified challenges inherent in identifying the 10 most important priorities among a large number of potential candidates. Participants indicated that having to select only 10 priorities from the original top 30 shortlist was “overwhelming” (ID13, policymaker). Some related this challenge to the implications of classifying a single priority in the top 10, as only the top 10 priorities contributed to a final published list. Participants generally regarded all priorities as important, and many thought it seemed arbitrary which priorities ultimately made the final top 10. For example,

In the end, being 11 or 12 really stinks, because it's only the top 10 that really get looked at, I would assume. And so anything that you thought was important and that maybe got fairly close but didn't get up there, I can see why you'd suddenly want to really push to get it into that 10. (ID4, patient)

There was a lot of give and take, and you could've easily taken out three or four or five and put in three or four or five different ones, but it would've just been as acceptable. It was hard narrowing it down to 10, that's for sure. (ID7, caregiver)

Despite these challenges, the process for identifying and ranking CKD research priorities was perceived as systematic and rigorous. One HCP said that she felt the participants “knew exactly what they needed to do at every stage and they knew how to effectively participate” (ID20), and for one policymaker, that this multi‐stakeholder approach to research prioritization “does seem like a pretty solid way of moving forward” (ID9).

Across all interviews, the in‐person workshop was considered the preferred method for identifying research priorities. For example, one workshop patient speculated that there “was a much better rapport” (ID10) in person, and another questioned whether or not “you would get that type of interaction in an online forum” (ID5). The three HCP participants who had experience with both in‐person and wiki formats for research prioritization all expressed a preference for the in‐person format. In general, wiki participants appeared to be discouraged by the low participation rates and lack of justification for rankings through the online chat feature. One wiki participant related the lower engagement in this format to the lack of opportunity for team building and rapport:

The discretionary decision to participate or not is based on a commitment that involves relationships. And to me, there was no relationship in this thing. I didn't know who the other people were, I couldn't figure out their role… I couldn't maintain a sense of the overall flow of thought as the thoughts progressed. (ID11, policymaker)

Some participants acknowledged potential advantages to the wiki, such as convenience and comfort expressing one's opinion. Further, many participants remarked upon the similarity between the final workshop and wiki top 10 lists.^12^ One patient, who expressed being sceptical initially, subsequently suggested the overlap in the two groups' priorities “proves the concept that there is some validity to this wiki stuff” (ID12).

### 3.3 Application and applicability of priorities

Participants discussed their understanding of what had happened with the priorities since completion of the priority‐setting project, such as funding applications, on‐going initiatives and publications. Whereas some participants recalled learning about on‐going research related to the final priorities, several participants across all stakeholder groups indicated they were unaware of the project's impact. This led to discussions around what participants considered appropriate with respect to communication and follow‐up. Although all participants were genuinely interested in the outcomes of the priority‐setting project, one caregiver “did not look at [the lack of on‐going communication] as a bad thing” (ID6). In contrast, one policymaker regarded the failure to communicate a project's impact as a “silo symptom” (ID11). Some participants indicated their preference for on‐going feedback on the impact of their engagement, which, for one patient, would have reassured him that “what we did is making a difference” (ID10). Follow‐up communication may have also facilitated closure for participants, as one policymaker described:

I think people need a sense of closure if the project has ended… I can understand someone who isn't used to engaging in this kind of thing, and this would have been a new and different initiative for them, to have been very engaged and then to suddenly have it all stop would have maybe been a bit difficult. (ID9)

Interestingly, some participants had referred to the research priorities in the care of their own or their patients' CKD. For one patient, reference to the priorities “validated we're on the right track” (ID22) with respect to his CKD care, and for one HCP, recognition of the importance patients placed on symptoms led him to “temper my education a bit” (ID8) and focus more on patient priorities. Some HCPs also described the circumstances under which they had referred to the priorities in research, such as in generating ideas for local initiatives or “when I've been applying for grants” (ID18, HCP). However, some participants questioned the feasibility of the priorities as research questions and contrasted them with priorities for patient care:

From what I understand, it's [CKD] not going away, people just have to manage it. And it's very important to manage it the best way people can. From a patient, caregiver kind of aspect, I thought the quality of life ones [priorities] were important, right? But maybe not in the typical research vein. (ID7, caregiver)

I guess I look at the list as being rather vague and general. So, for example, if you were a funding agency and you were shown this list and then shown 20 research projects, and you were only able to fund five, I'm not sure this list would be helpful. (ID15, HCP)

Regardless, participants recognized the importance of patients and other stakeholders collectively identifying priorities for CKD research. In the context of research funding allocation, one policymaker summarized her views on priorities' alignment among stakeholders:

I think we can learn a lot from them [patients/caregivers]. I think it does behoove us in a certain way to listen to them and what their priorities are and try to make some attempts to fund along those lines. (ID9)

## 4 DISCUSSION

In this study, we found that stakeholders from a CKD research priority‐setting exercise remained in agreement with the priorities identified 2 years previously, even though their individual priorities may have changed in the interim in relation to their own experiences. Despite a perceived distinction between the priorities of patients/caregivers and those of HCPs/policymakers, the top priority (“interventions to slow CKD progression”) was still felt to be most important across stakeholder roles. Further, participants acknowledged the broad potential reach of the priorities and attributed their perceived validity to the systematic, inclusive and rigorous priority‐setting process. These findings support prioritization exercises such as that of the JLA, which aim to address mismatches between priorities of researchers and those with lived experience of a health condition.^6^, ^28^ However, participants in our study wondered whether some perspectives, in particular those from socio‐demographically disadvantaged groups, may have been underrepresented in this forum and thus at the relevance of the priorities to the broader CKD population.

To our knowledge, no other study has systematically addressed the ways in which stakeholders' research priorities, and those of patients in particular, may change over time. Other research has explored how patients' health‐care priorities^29^, ^30^ and value systems^31^ may change as a consequence of new medical diagnoses and/or multimorbidity. In the area of public deliberation, the views of citizens' jury participants may be influenced by their jury experience, and in one study, these views were retained upon questionnaire several weeks later.^32^ Although no similar reports exist in the health research prioritization literature, our study supports the intermediate‐ to long‐term stability of public views that are informed by deliberative processes. However, it remains unclear whether or how priorities might change over a longer time period and what the implications of such a shift may be. This is an important consideration given the increasing dedication of research funding and resources to priorities identified by stakeholders, such as patients, and that priority‐driven projects can take several years to undertake, disseminate and implement in practice. Although no established, system‐level processes exist for responding to longer‐term changes in priorities, this study provides an important first step in contrasting changes in individual priorities with the perceived stability and relevance of group priorities over time.

The final step in the JLA approach is rooted in deliberative methods whereby participants are encouraged to discuss their views, consider different perspectives and arrive at a reasoned group decision (ie final top 10 priorities).^11^, ^33^ Participants in our study appeared to view this process as systematic, inclusive and equitable through all of its stages; they perceived their interactions as respectful, denied tensions between stakeholder groups and suggested that consensus was achieved. However, participants expressed a preference for the in‐person format over the online wiki‐based platform for the final prioritization step due to perceived barriers to communication and rapport online. The views of participants in this study endorse identified barriers to online collaborative writing application use in health‐care settings, such as tool unfamiliarity, time constraints, technical concerns and frustration by low participation.^34^ Familiarity and relationships among group members have been identified as an important element underlying effective stakeholder engagement,^35^ and in this setting, it could be strengthened by allowing participants to meet informally beforehand (eg via teleconference), as one of our study's participants suggested.

Prioritization exercises such as that of the JLA are being used to identify and prioritize research topics on an international scale and across a number of health conditions. To date, more than 60 JLA priority‐setting partnerships have identified priorities for conditions such as asthma, schizophrenia and urinary incontinence, among others, which have been used to inform research programmes worldwide.^11^ The JLA is making a concerted effort to track the impact of published priorities resulting from its priority‐setting partnerships. However, this can be challenging in the light of the public availability of the priorities and the fact that researchers can interpret and apply them as they choose.^18^ A scoping review assessed the extent to which completed or on‐going clinical research aligned with stakeholder‐identified priorities from a JLA exercise for those on or nearing dialysis.^9^ Less than one‐fifth of included studies addressed topics consistent with the top 10 priorities, and most focused on cardiovascular health while neglecting other priority areas. This review was published ahead of Canada's largest investment in research to improve care for persons with kidney disease, the Canadians Seeking Solutions and Innovations to Overcome Chronic Kidney Disease (Can‐SOLVE CKD) Network.^13^ The research activities within this network were derived in large part from patient and stakeholder priorities identified through JLA partnerships across the CKD spectrum.

Although participants in our study seemed to agree on the importance of the final priorities, some raised concerns about their scope, clarity and feasibility. To some HCPs, the research priorities seemed too vaguely worded and/or broad in scope to permit their operationalization; likewise, patient/caregiver participants commented on the difficulty in ranking priorities that appeared to address similar issues or use similar wording. Further, we observed that while patients and caregivers prioritized areas related to their care or personal experiences, HCPs questioned the feasibility and uncertainty of some as *research* priorities. In a reflective piece, Madden and Morley^36^ discussed challenges to defining research priorities from a pressure ulcer JLA partnership, including adequately capturing the expressed idea underlying each priority without being unnecessarily detailed. Finding this balance between generality and specificity in defining the priorities is imperative to facilitating their subsequent application as research questions.^36^

The limitations of this study relate primarily to issues of study design and sampling. First, as 2 years had elapsed since the CKD priority‐setting project, participants may not have recalled details about the prioritization processes or their individual priorities. However, we focused on participants' long‐term views and subsequent experiences, and we provided participants the final priorities' lists in advance of the interviews to prompt discussion. Second, we acknowledge that in this study, participants discussed their individual approaches to research prioritization, whereas prioritization at the time of the priority‐setting partnership occurred through group deliberation and consensus. We also did not collect individually ranked priorities at the time of the workshop/wiki. However, elicitation of participants' current priorities was not intended for comparative purposes, but rather to encourage reflection and stimulate discussion on why and how they prioritize certain areas over others. Third, participants were heterogeneous in that they were from different stakeholder groups and had participated in different aspects of the project, including some who took part in more than one step (eg some steering committee members also participated in the workshop). As we aimed to capture both the diversity in perspectives and common patterns across this variation, we intentionally sampled across these parameters and feel this contributed to the collection of rich data. Despite the variation in type and extent of engagement, participants expressed a perceived validity of the JLA priority‐setting process overall and shared the view that engagement through the online wiki‐like platform did not allow for the same sense of partnership and familiarity as direct, interpersonal contact. Lastly, we acknowledge that our findings may be context specific and that participants' priorities and experiences with research prioritization may not reflect those in different settings. Further, it is possible that those who participated in this study held strong views that may have influenced the original priorities and that these views may have differed from those of the broader CKD stakeholder population or eligible individuals who declined an interview. The inclusion of all relevant perspectives remains a challenge to stakeholder‐engaged research, and strategy for engaging representative samples and encouraging contributions across participants is an area of study that requires further attention. Nevertheless, the increasing engagement of patients as stakeholders in research prioritization across health‐care disciplines supports the relevance of our identified themes to other engagement and prioritization contexts.

Findings from our study have implications for health research that engages patients and other stakeholders, particularly in identification of research priorities. We have characterized the circumstances under which CKD stakeholders' individual priorities may change over time, even though participants felt the groups' decisions remained valid and reflective of the deliberative process. However, the implications of the resulting priorities must be carefully considered in relation to those who were included in the prioritization process. The fact that certain populations may be underrepresented in such exercises (eg those with advanced illness or from marginalized groups) highlights the need to adopt inclusive strategies for engagement that ensure balanced representation of all relevant perspectives.^37^ Further, most participants in our study were unaware of the outcomes of the CKD priority‐setting project, thus raising questions about how to optimize stakeholder engagement and expectations for follow‐up upon study completion. In a case study of a UK Research and Development consortium, the most important concern raised by public volunteers was their desire for feedback on the value of their contributions and the research outcomes.^38^ Others considering future research prioritization partnerships should define strategies at the outset for dissemination of the priorities and their impact to participants. Lastly, participants' experiences with different prioritization formats (ie in person vs online) highlight the need for future study on alternative approaches to research engagement, particularly for those with health limitations who may not otherwise be able to participate. An acceptable format must not only permit the research task at hand but also facilitate rapport and communication among participants.

## 5 CONCLUSION

Two years after engaging in a CKD research prioritization exercise, stakeholders remained in agreement overall with the previously identified priorities. Even though their individual experiences may have influenced their views on the priorities, participants suggested that group deliberation and consensus were key elements supporting the prioritization process and credibility of identified priorities. Participants emphasized communication, feedback and rapport among participants and the research team, thus highlighting opportunities for future work to enhance the experience of stakeholders collaborating in research.

## CONFLICT OF INTEREST

The authors have no conflict of interests to disclose.
