# Supplementary material for: Long‐term views on chronic kidney disease research priorities among stakeholders engaged in a priority‐setting partnership: A qualitative study
Source: Health Expect. 2018 Aug 15;21(6):1142–9. doi: 10.1111/hex.12818 (PMC6250874; doi:10.1111/hex.12818)
Supplement: Supplementary file 1 [file HEX-21-1142-s001.docx]

**Appendix 1.** Participants from CKD priority-setting partnership eligible for inclusion in this study

| **Stakeholder Role** | **Steering Committee Only**  **(N=2)** | **Steering Committee and Workshop (N=6)** | **Workshop Only**  **(N=19)** | **Wiki**  **(N=26)** |
| --- | --- | --- | --- | --- |
| Patient^*^ | 1 | 2 | 8 | 10 |
| Caregiver |  | 1 | 5 | 9 |
| Healthcare Professional | 1 | 2^†^ | 5 | 6 |
| Policymaker |  | 1 | 1 | 1 |

^*^ Does not include 3 patients who had died since completion of the priority-setting project

^†^ One HCP on the steering committee/workshop was a participant, one was a facilitator

**Appendix 2.** Interview guide

| **Part 1: Engagement in CKD Priority Setting Project** |
| --- |
| Tell me about your experiences with research before this particular project, if any.   - Prompt: What was your role in this previous research? How did you become interested in getting involved in research? What type of training do you have in research, if any? |
| Can you tell me about your experience in this project to determine the top 10 CKD research priorities?   - Prompt: How did you learn about this project? Why did you participate? - Prompt: What was your role in the project? How involved did you feel? How did you feel working alongside others with different backgrounds? What did you think of the format? |
| **Part 2: CKD Research Priorities** |
| Tell me your thoughts on the final top 10 priorities from your group <<workshop/wiki>>.   - Prompt: How did you feel about the priorities then? How would you rank them now? Why? - Prompt: When you’re thinking about priorities for kidney research, what types of things factor into your decisions (i.e. how do you weigh them)? |
| What do you know of what has happened with the final priorities since then?   - Prompt: What research projects or initiatives have you heard about using these priorities? How do you feel about the communication with the research team since this project? |
| **Part 3: Long-Term Perceived Engagement** |
| [Patients/caregivers] How would you describe your/patient’s kidney disease now? General health?   - Prompt: In what ways is this similar to or different from when you participated in the project? How do you look after your/patient’s kidney disease?   [HCPs/policymakers] How would you describe your current scope of practice/position?   - Prompt: In what ways is this similar to or different from when you participated in the project? |
| Reflecting back, what did you take away from your experience with the priority setting project?   - Prompt: How do you think about health research now? Did you learn anything about kidney disease from this experience? Explain. |
| Is there anything you are doing differently with respect to your/patient’s kidney disease [patients/caregivers] or CKD care/research/policy [HCPs/policymakers] since participating in this project? Explain.   - Prompt [patients/caregivers]: Are you managing your/patient’s CKD differently now? If yes, how? Tell me about your experiences with research since then, if any. - Prompt [HCPs]: Are you caring for people with CKD differently now? If yes, how? - Prompt [policymakers]: Has your involvement in this project influenced how you determine policy or make decisions related to CKD care? If yes, how? - Tell me about your experiences with research since then, if any. [HCPs/policymakers] To what extent have you engaged with patients in your research/work since? |
| **Part 4: Other General Engagement** |
| When you think about engaging in research with patients, caregivers, HCPs and policymakers, what are some things that are important to you?   - Prompt: How can we as researchers make it a better experience for you? |
| How did you use technology to take part in this project (e.g. email, wiki, online communication)? |
| How did the research team show their appreciation for your time and contributions to this work?   - Prompt: How would you have liked to have been acknowledged? How do you feel about compensating non-researchers (i.e. patients/families) for participating in research? |
| **Part 5: Concluding Questions** |
| Is there anything you would like to add about your involvement in the CKD priority setting project that we haven’t already discussed? Do you have any other thoughts about working together with patients and others affected by kidney disease in research? |
